# Identifying Common Genetic Etiologies Between Inflammatory Bowel Disease and Related Immune-Mediated Diseases

**DOI:** 10.3390/biomedicines12112562

**Published:** 2024-11-08

**Authors:** Xianqiang Liu, Dingchang Li, Yue Zhang, Hao Liu, Peng Chen, Yingjie Zhao, Piero Ruscitti, Wen Zhao, Guanglong Dong

**Affiliations:** 1 Medical School of Chinese PLA, Beijing 100853, China; dr_liuxianqiang@163.com (X.L.);; 2 Department of General Surgery, The First Medical Center, Chinese PLA General Hospital, No. 28 Fuxing Road, Haidian District, Beijing 100853, China; 3 School of Medicine, Nankai University, Tianjin 300071, China; 4 Rheumatology Unit, Department of Biotechnological and Applied Clinical Sciences, University of L’Aquila, 67100 L’Aquila, Italy

**Keywords:** inflammatory bowel disease, GWAS, genetics, MTAG, immune-mediated diseases, IL-1, autoinflammation

## Abstract

Background: Patients with inflammatory bowel disease (IBD) have an increased risk of developing immune-mediated diseases. However, the genetic basis of IBD is complex, and an integrated approach should be used to elucidate the complex genetic relationship between IBD and immune-mediated diseases. Methods: The genetic relationship between IBD and 16 immune-mediated diseases was examined using linkage disequilibrium score regression. GWAS data were synthesized from two IBD databases using the METAL, and multi-trait analysis of genome-wide association studies was performed to enhance statistical robustness and identify novel genetic associations. Independent risk loci were meticulously examined using conditional and joint genome-wide multi-trait analysis, multi-marker analysis of genomic annotation, and functional mapping and annotation of significant genetic loci, integrating the information of quantitative trait loci and different methodologies to identify risk-related genes and proteins. Results: The results revealed four immune-mediated diseases (AS, psoriasis, iridocyclitis, and PsA) with a significant relationship with IBD. The multi-trait analysis revealed 909 gene loci of statistical significance. Of these loci, 28 genetic variants were closely related to IBD, and 7 single-nucleotide polymorphisms represented novel independent risk loci. In addition, 14 genes and 514 proteins were found to be associated with susceptibility to immune-mediated diseases. Notably, IL1RL1 emerged as a key player, present within pleiotropic genes across multiple protein databases, highlighting its potential as a therapeutic target. Conclusions: This study suggests that the common polygenic determinants between IBD and immune-mediated diseases are widely distributed across the genome. The findings not only support a shared genetic relationship between IBD and immune-mediated diseases but also provide novel therapeutic targets for these diseases.

## 1. Introduction

Inflammatory bowel disease (IBD), which includes both Crohn’s disease and ulcerative colitis, is a chronic inflammatory disorder. It arises from an abnormal immune reaction to intestinal bacteria in individuals with a genetic predisposition, leading to recurring and damaging inflammation of the gastrointestinal tract [1]. Millions of individuals have IBD worldwide, with approximately 2.2 million diagnosed cases in Europe alone [2]. Immune-mediated diseases frequently co-occur within families, with individuals with one condition having an increased risk of other conditions [3,4]. Common susceptibility loci identified in various immune-mediated diseases suggest a shared genetic basis involving polygenic susceptibility genes [5,6]. Comorbidities and associations between inflammatory bowel disease and immune-mediated diseases have been widely reported. Genome-wide association studies (GWASs) have validated a genetic relationship between IBD and several immune-mediated diseases, including celiac disease [7], rheumatoid arthritis (RA) [8], ankylosing spondylitis (AS) [9], and primary sclerosing cholangitis [10]. Numerous signaling pathways are involved in this relationship, with tumor necrosis factor (TNF) and nuclear factor kappa-light-chain-enhancer of activated B cells (NF-κB) playing a crucial role in inflammation and other immune responses [11,12].

Despite significant advancements in understanding these relationships, studies focusing on single diseases may overlook key genetic loci and regulatory mechanisms. Therefore, multi-trait analysis is important for expanding the phenotypic spectrum of research, identifying risk loci, and investigating the shared genetic etiological factors between diseases [13]. The shared genetic causes may indicate potential pleiotropy, acting as genetic confounding factors between traits [14,15]. Therefore, cross-trait analysis based on GWAS data has been proposed to identify pleiotropic genes or loci across multiple traits [16,17]. These pleiotropic loci may serve as valuable targets for preventing or treating multiple diseases simultaneously.

**Figure 1:**
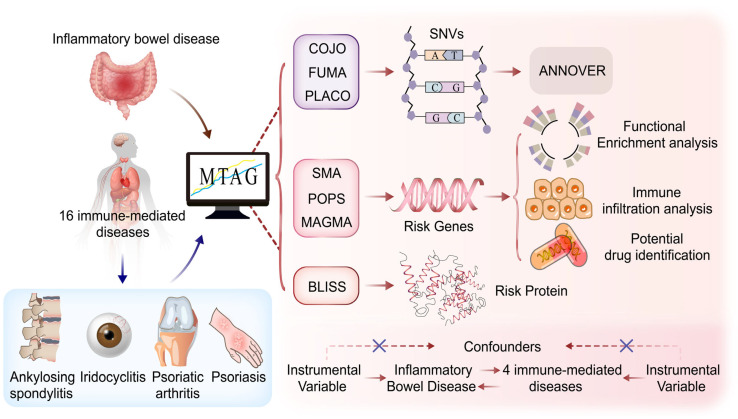
The overall study design. LDSC analysis was first conducted on IBD and 16 immune-mediated diseases, revealing a genetic correlation between IBD and conditions such as AS, psoriasis, iridocyclitis, and PsA. Following this, MTAG analysis was performed, and the indings were elaborated at the levels of single-nucleotide variants (SNVs), genes, and proteins. The figure was created using Adobe Illustrator. AS, ankylosing spondylitis; PsA, psoriatic arthritis; LDSC, linkage disequilibrium score regression; POPS, polygenic priority score; MAGMA, multi-marker analysis of genomic annotation; COJO, conditional and joint analysis; BLISS, Biomarker Imputation from Summary Statistics; SMR, Summary-based Mendelian randomization; MR, Mendelian randomization.

In this pleiotropic GWAS, summary data from large-scale GWASs on IBD (International Inflammatory Bowel Disease Consortium and FinnGen database) and 16 immune-mediated diseases were utilized. Various statistical methods (PLACO, MAGMA, POPS, SMR, and BLISS) were used to assess pleiotropic associations between IBD and immune-mediated diseases based on single-nucleotide variants (SNVs), gene levels, protein levels, and biological pathways to identify shared genetic etiological factors. Furthermore, gene set variation analysis (GSVA) was performed based on the transcriptomic profiles of patients with IBD to establish a pleiotropic gene score, and the relationship between this score and immune cell infiltration was subsequently analyzed to subtype IBD patients. We hope that this study will contribute to a better understanding of the genetic structure and potential therapeutic mechanisms of IBD. Figure 1 depicts the comprehensive design of this study.

## 2. Materials and Methods

### 2.1. Collection of GWAS Summary Data

GWAS summary statistics were obtained from publicly available datasets of European ancestry. GWAS summary data on IBD were extracted from the International IBD Genetics Consortium [18], which includes 12,882 patients and 21,770 control individuals, and a FinnGen database (Finn-IBD), which includes 9083 patients and 392,974 control individuals. Additionally, data on 16 prevalent immune-mediated diseases were obtained from the FinnGen database [19], including AS, psoriasis, iridocyclitis, psoriatic arthritis (PsA), celiac disease, sarcoidosis, primary sclerosing cholangitis, multiple sclerosis, immune-mediated hyperthyroidism, RA, type 1 diabetes mellitus, hypothyroidism, adult-onset Still’s disease, hidradenitis suppurativa, gout, and systemic lupus erythematosus. Based on data availability, these 16 diseases were chosen across an inflammatory spectrum from autoinflammatory to autoimmune diseases as two-end stages and “intermediate” phenotypes according to a continuum of different pathologic conditions associated with a deregulated and aberrant immune response [20]. Data aligning with the GRCh38 reference were converted to GRCh37 using the liftOver tool to ensure consistency [21].

The characteristics of the study population are outlined in Supplementary Table S1. To investigate the genetic composition of patients with IBD, quantitative trait loci (QTL) data were integrated, including plasma protein QTLs (pQTLs) and expression QTLs (eQTLs) from 54 specific samples (such as small intestine terminal ileum and Colon Sigmoid). Blood eQTL data were extracted from the expansive eQTLGen Consortium database, documenting single-nucleotide polymorphisms (SNPs) associated with various traits in 31,684 individuals [22]. Plasma pQTL data were extracted from the deCODE [23], ARIC, and UKBPP [24] database.

### 2.2. Assessment of Genetic Correlations Between IBD and Immune-Mediated Diseases

Linkage disequilibrium score regression (LDSC) was used to assess the genetic relationship between IBD and 16 immune-mediated diseases based on GWAS summary data from two IBD datasets. In particular, the 1000 Genomes Project data for European ancestry was harnessed for LD scores within these analyses [25]. In LDSC, no constraints were imposed on the intercept, as sample overlap influences only the intercept, leaving the regression slope and genetic correlation unaffected. The Benjamini and Hochberg (BH) correction was applied to mitigate the impact of multiple testing, with an FDR-*p* value < 0.05 confirming significant genetic associations. Immune-mediated diseases that showed positive results in both dataset (The International Inflammatory Bowel Disease Genetics Consortium and Finngen database) analyses were included in the subsequent analysis.

### 2.3. Meta-Analysis of GWASs

A meta-analysis was conducted to combine data from two sources of IBD. Using Metasoft, the heterogeneity index (I^2^) and *p*-value based on Cochran’s Q test (*P*_het) were calculated. In cases of heterogeneity (I^2^ ≥ 50 or *P*_het < 0.05), *p*-values from the random-effects model were prioritized [26,27].

### 2.4. Integrated Analysis of Traits of IBD and Associated Immune-Mediated Diseases

Based on the results of the abovementioned analyses, the multi-trait analysis of GWASs (MTAG) method was used to assess the phenotypes of immune-mediated diseases that exhibited a significant genetic correlation with META-IBD. The MTAG involves the use of the generalized inverse-variance weighted (IVW) approach to enhance statistical power and facilitate the identification of genetic relationships for each trait. Essentially, the MTAG processes the GWAS summary data of individual traits to produce trait-specific effects for shared SNPs while integrating LDSC to adjust for sample overlap between related traits [28]. In this process, the GWAS data of immune-related disease phenotypes with confirmed genetic correlations to IBD were integrated to generate MTAG-IBD.

### 2.5. Identification of Genetic Risk Factors for IBD

#### 2.5.1. Identification of Independent Risk Loci

To further elucidate the genomic risks associated with IBD, distinct independent signals within genomic loci linked to MTAG-IBD were examined. This was achieved through conditional and joint association analyses using the stepwise model selection framework provided by GCTA–conditional and joint analysis (COJO) [29]. The analysis focused on multi-allelic variants displaying significant associations (*P*.mtag < 5 × 10^−8^) within recognized genomic risk loci, with additional signals being validated based on a joint *p*-value threshold of <5 × 10^−8^. This investigation utilized the European ancestry reference cohort dataset from the third phase of the 1000 Genomes Project [25]. For pairs of traits showing significant genetic correlation or overlap, pleiotropic analysis under the composite null hypothesis (PLACO) was employed to detect potential pleiotropic SNVs. This analysis computed squared Z scores for each variant, and SNPs with exceedingly high Z values (>80) were excluded. Subsequently, the hypothesis of no pleiotropy was assessed using the intersection–union test method with a horizontal α, leading to determining the final pleiotropic *p*-values. Significant pleiotropic variants were identified as SNVs with *p*-values < 5 × 10^−8^. Following the PLACO results, the identified loci were mapped to neighboring genes to investigate the shared biological mechanisms underlying these pleiotropic loci. Additional annotation was performed using the Functional Mapping and Annotation (FUMA) platform [30]. Strict criteria were applied, setting the maximum *p*-value for lead SNVs at <5 × 10^−8^ while adopting a more inclusive significance threshold of *p* < 0.05. Independent SNV identification was based on an r^2^ threshold of less than 0.6, with lead SNVs requiring an r^2^ of less than 0.1 within a 1 Mb radius. The criteria for defining genomic risk loci included merging regions with lead SNVs less than 250 kb apart. SNVs validated through COJO and FUMA analyses were classified as risk variants for COPD. These variants were annotated using ANNOVAR [31], and their potential deleterious impact was evaluated through combined annotation-dependent depletion (CADD) scores, with values exceeding 12.37 suggesting a higher likelihood of harmful effects [32]. RegulomeDB (RDB) [33] provides a classification score ranging from 1a to 7, indicating the regulatory function of SNPs based on eQTLs and chromatin marks. A score of 1a represents the strongest biological evidence for the SNP as a regulatory element.

#### 2.5.2. Genetic Insights into IBD

In a comprehensive analysis targeting the genetic underpinnings of IBD, three distinct strategies were implemented. For each approach, *p*-values were corrected for the false discovery rate (FDR) using the BH method, with genes displaying an FDR < 0.05 and those consistently identified by three approaches being deemed significantly associated risk genes. The multi-marker analysis of genomic annotation (MAGMA) and polygenic priority score (POPS) were applied to identify and prioritize relevant genes [34,35]. The gene-centric analysis conducted in the MAGMA was derived from an extensive catalog of protein-coding genes, and when combined with the POPS, it enabled a more refined gene prioritization strategy. This methodology integrated summary GWAS data with comprehensive expression datasets and biological pathways, establishing a POPS threshold greater than 1 for gene selection.

The Summary-based Mendelian randomization (SMR) approach was simultaneously applied, utilizing the MTAG-IBD and eQTL dataset summary statistics to identify genes whose expression levels are associated with complex traits driven by pleiotropy. SMR uses SNPs as instrumental variables to test the causal effect of the exposure on the outcome [36]. The accompanying Heterogeneity in Dependent Instruments (HEIDI) test was also employed to assess whether the causal effect detected by SMR was due to linkage disequilibrium (*p*-HEIDI < 0.01). Consequently, genes with an FDR < 0.05 and *p*-HEIDI > 0.01 were considered potential susceptibility genes associated with the genetic risk underlying MTAG-IBD.

Phenotypic and genomic enrichment analyses were performed to assess the biological relevance of genes associated with IBD. Phenotypic enrichment analysis was performed using the Mammalian Phenotype Ontology from Mouse Genome Informatics (MGI) [37], and the specificity of mammalian phenotype (MP)-related genes was compared with that of non-MP-related genes. The genomic enrichment analysis was performed using data from the Kyoto Encyclopedia of Genes and Genomes (KEGG) pathways and Gene Ontology (GO) pathway databases [38,39].

#### 2.5.3. Proteomic Insights into IBD

To unravel the complex proteomic profiles linked with IBD and immune-mediated diseases, this study utilized the “Biomarker Imputation from Summary Statistics (BLISS)” technique [40]. BLISS introduces a novel approach for developing protein interpolation models directly from summary-level pQTL data, thereby enabling the creation of comprehensive European PWAS models. This was accomplished by employing extensive pQTL information from large repositories, including the UKB, deCODE, and ARIC studies. In this research segment, the BH procedure continued to be applied to ascertain the FDR of *p*-values. Proteins exhibiting an FDR of less than 0.05 were recognized as significant risk proteins, highlighting their potential role in the pathophysiology of IBD and related immune-mediated diseases.

### 2.6. Construction of Drug–Gene Interaction Networks

The Drug–Gene Interaction Database (DGIdb, http://www.dgidb.org/, accessed on 28 May 2024) [41] was used to predict FDA-approved drugs interacting with pleiotropic genes. Subsequently, Cytoscape software (version 3.9.1) was used to construct a drug–gene interaction network based on the predictions.

### 2.7. Two-Sample MR

The widely recognized instrumental variable technique, MR analysis, was employed to discern causal relationships between IBD and four immune-related diseases. SNPs associated with exposure served as instruments [42,43], while data derived from GWAS summaries were utilized to pinpoint variants correlated with IBD and four immune-related diseases, adhering to a *p*-value threshold of <5.0 × 10^−8^. Additionally, to account for linkage disequilibrium, a clumping procedure was employed on these SNPs using specific parameters (kb = 10,000, r^2^ = 0.001). To ensure the robustness of the instrumental variables, the coefficient of determination (r^2^) and F-statistic were computed, incorporating only those SNPs with F-values exceeding 10. Furthermore, the MR-PRESSO method, employing 1000 iterations, was utilized to identify outliers [14]. LDlink facilitated the exclusion of confounding SNPs, and the identified outliers were subsequently removed for reassessment.

### 2.8. GSVA and Immune Cell Infiltration Analysis

To assess the role of pleiotropic genes in the immunophenotyping of IBD patients, GSVA analysis was performed to evaluate immune cell infiltration; mRNA expression data were extracted from three datasets (GSE75214, GSE3365, and GSE87466) from the Gene Expression Omnibus (GEO) database [44], including 344 patients with IBD and 85 healthy individuals. Risk-related genes were quantified using the single-sample gene set enrichment analysis (ssGSEA) algorithm. Based on the ssGSEA outcomes, patients in the IBD cohort were classified with high or low immune infiltration [45].

## 3. Results

### 3.1. Genetic Relationship Between IBD and Multiple Immune-Mediated Diseases

In investigating genetic correlations, we conducted LDSC analyses on IBD from two sources and 16 immune-mediated diseases. After applying BH correction, we identified significant genetic correlations between IBD from both sources and four immune-mediated diseases (AS, psoriasis, iridocyclitis, and PsA) (Supplementary Table S2). Subsequently, we integrated the GWAS summary statistics from both IBD sources to create a consolidated dataset (META-IBD), which included 9,749,028 SNPs. After excluding the complex MHC region, we identified 8033 statistically significant genetic loci.

### 3.2. Genetic Landscape of IBD Identified Through Multi-Trait Analysis

To further investigate the complex genetic architecture of IBD, we performed a multi-trait analysis using the META-IBD dataset and four immune-mediated diseases. Using the MTAG method, we generated an enhanced IBD dataset (MTAG-IBD) that included 8,247,248 SNPs. From this MTAG-IBD dataset, we identified 8657 significant SNPs. Notably, this analysis revealed 909 novel SNPs that had not been previously detected in individual IBD GWAS summaries or the META-IBD dataset.

### 3.3. Genetic Markers Associated with IBD

**Figure 2:**
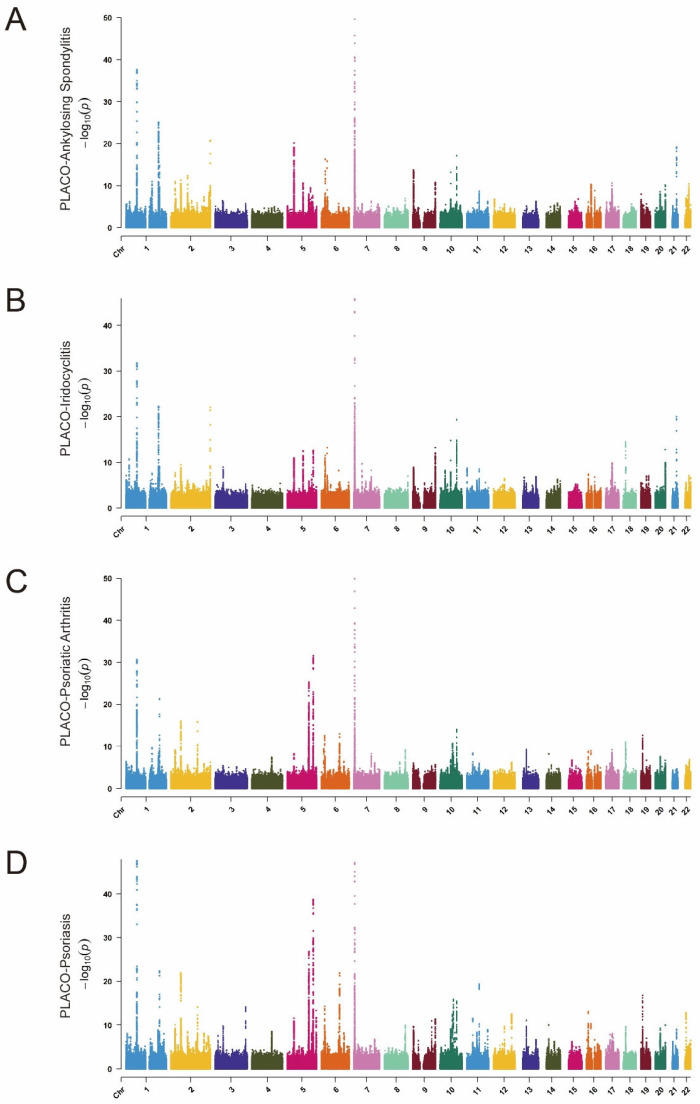
Manhattan plots of gene-based enrichments of PLACO associations with (**A**) ankylosing spondylitis, (**B**) iridocyclitis, (**C**) psoriatic arthritis, and (**D**) psoriasis. The *x*-axis shows chromosomal position, and the *y*-axis shows association *p*-values on a −log_10_ scale.

Using the advanced GCTA-COJO tool, we conducted conditional and joint association analyses on the MTAG-IBD dataset. Through this rigorous process, we identified 363 SNPs (Supplementary Table S3). Subsequently, the PLACO analysis identified 9344 SNPs as potential pleiotropic variants across four IBD–immune-mediated disease pairs (Figure 2A–D). Further analysis using the FUMA platform revealed 159 lead SNPs (Supplementary Table S4). Notably, 38 SNPs (28 unique) were consistently identified in both the COJO and PLACO analyses, establishing them as independent genetic risk loci for IBD–immune-mediated diseases. Utilizing the ANNOVAR tool for further detailed gene annotation, we identified two variants—rs11209026 (IL23R) and rs1990760 (IFIH1)—located within the exonic regions. Notably, both of these variants have CADD scores exceeding 12.37. This suggests the potential pathogenic effects of these single-nucleotide variants (SNPs).

### 3.4. Genes Associated with the Risk of IBD

**Figure 3:**
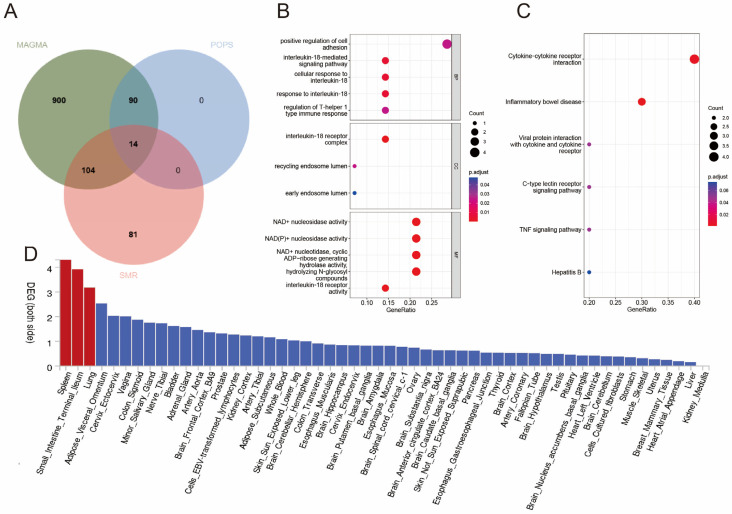
Enrichment analysis for identified pleiotropic genes. (**A**) The Wayne diagram showing POPS, MAGMA, and SMR pleiotropic gene screening. Significant types of pathways based on GO (**B**) and KEGG enrichment analyses (**C**). BP, biological process; CC, cellular component; MF, molecular function; KEGG, Kyoto encyclopedia of genes and genomes pathway. (**D**) Enrichment of differentially expressed genes among all identified pleiotropic genes across 54 GTEx tissues. The *y*-axis shows the *p*-values with a scale of −log_10_. The bars in red represent significant enrichment with Bonferroni adjustment for multiple-hypothesis testing.

The MAGMA revealed 1426 genes associated with immune-mediated disease risk SNPs (Supplementary Table S5). Subsequently, POPSs revealed 137 (104 unique) genes (POPSs > 1) potentially associated with the risk of IBD (Supplementary Table S6). SMR analysis showed that 14 of the 104 genes were associated with immune-mediated diseases (Figure 3A) (Supplementary Table S7). The analysis of tissue-specific gene expression revealed that disease–gene associations for pleiotropic genes were predominantly concentrated in the spleen and small intestine terminal ileum compartments (Figure 3D). Genomic enrichment analysis indicated that the risk-related genes were significantly enriched in biological processes related to the active regulation of inflammatory responses and immune effects, including positive regulation of leukocyte adhesion. In addition, the genes were enriched in Th17 cell differentiation, cytokine–cytokine receptor interactions, and the c TNF signaling pathway (Supplementary Table S8) (Figure 3B,C). These results suggest that the risk-related genes contributed to the pathogenesis of IBD and associated immune-mediated diseases. Phenotypic enrichment analysis showed that the genes were significantly enriched in two biological processes, including the immune system and hematopoietic system, demonstrating the complex nature of IBD and its systemic impacts (Supplementary Table S9).

### 3.5. Proteins Associated with the Risk of IBD and Associated Immune-Mediated Diseases

Based on the BLISS method combined with MTAG-IBD data, we evaluated 2923, 4953, and 4428 plasma proteins in the UKB, ARIC, and deCODE cohorts, respectively. The results revealed 514 proteins associated with the risk of IBD and associated immune-mediated diseases (Supplementary Table S10). KEGG enrichment analysis of these risk-associated proteins revealed significant enrichment in pathways such as cytokine–cytokine receptor interaction, JAK-STAT signaling, and TNF signaling (Supplementary Table S11). In particular, IL1RL1 was found to be present both within the pleiotropic genes and across the aforementioned three protein databases, suggesting a possible common thread and a potential therapeutic target.

### 3.6. Predicted Drug–Gene Interactions

**Figure 4:**
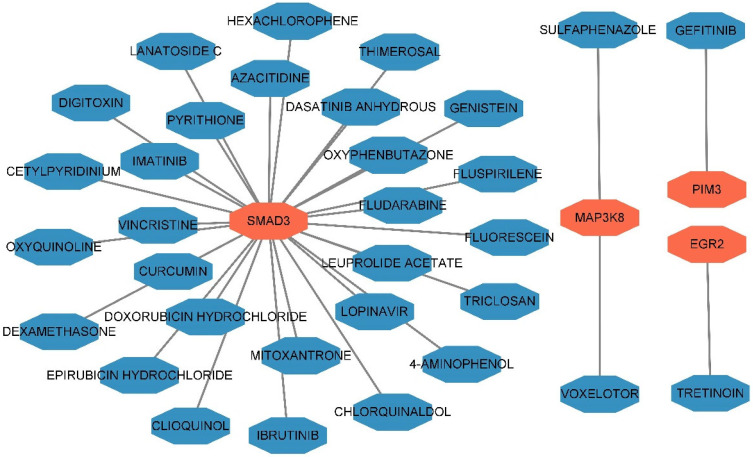
The drug–gene interaction network of the annotated genes through DGIdb. The genes are highlighted in red, whereas the drugs are highlighted in blue.

Cytoscape was used to construct a drug–gene interaction network (Figure 4 and Supplementary Table S12). A total of 32 interactions were observed between 32 small-molecule drugs and 4 pleiotropic genes (MAP3K8, EGR2, PIM3, and SMAD3), which may represent novel therapeutic targets for IBD.

### 3.7. Results of Two-Sample MR

**Figure 5:**
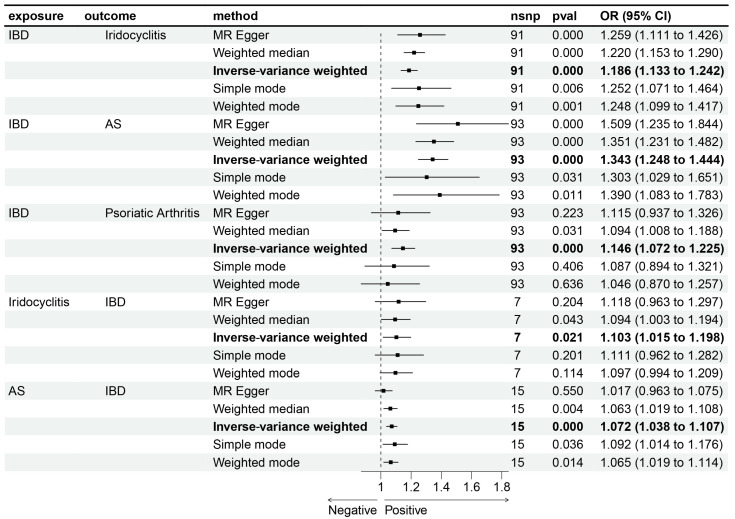
Bidirectional Mendelian randomization (MR) analyses between META-IBD and 4 immune-mediated diseases (ankylosing spondylitis, psoriasis, iridocyclitis, and psoriatic arthritis). Estimates and 95% confidence intervals (CIs) are shown using square plots and error bars.

**Figure 6:**
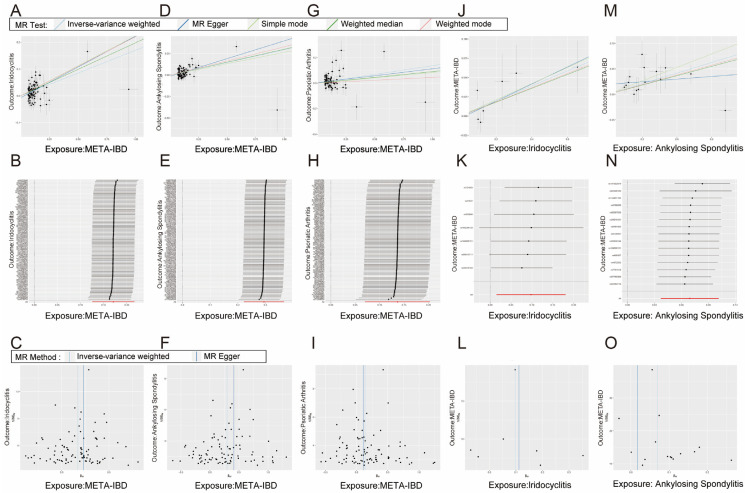
The scatter plot (**A**,**D**,**G**), sensitivity analysis (**B**,**E**,**H**), and funnel plot (**C**,**F**,**I**) of the effect of ankylosing spondylitis, psoriasis, and iridocyclitis on META-IBD. The scatter plot (**J**,**M**), sensitivity analysis (**K**,**N**), and funnel plot (**L**,**O**) of the effect of META-IBD on iridocyclitis and ankylosing spondylitis. In the scatter plot, five methods were employed to describe the direction of causality: IVW, Weight median, Simple Mode, Weighted Mode, and MR Egger.

Bidirectional MR was performed to investigate the potential causal relationship between IBD and immune-mediated diseases. The results indicate a potential causal relationship between META-IBD and iridocyclitis [OR: 1.19; 95% CI: 1.13–1.24], AS [OR: 1.34; 95% CI: 1.25–1.44], and PsA [OR: 1.15; 95% CI: 1.07–1.22], with increased incidence rates. Reverse MR demonstrates that iridocyclitis [OR: 1.10; 95% CI: 1.02–1.20] and AS [OR: 1.07; 95% CI: 1.04–1.11] have an inverse causal relationship with the incidence of META-IBD (Figure 5). Scatter plots, funnel plots, and leave-one-out sensitivity analysis are represented in Figure 6 (Supplementary Tables S13 and S14).

### 3.8. Identification and Characterization of IBD Subtypes

**Figure 7:**
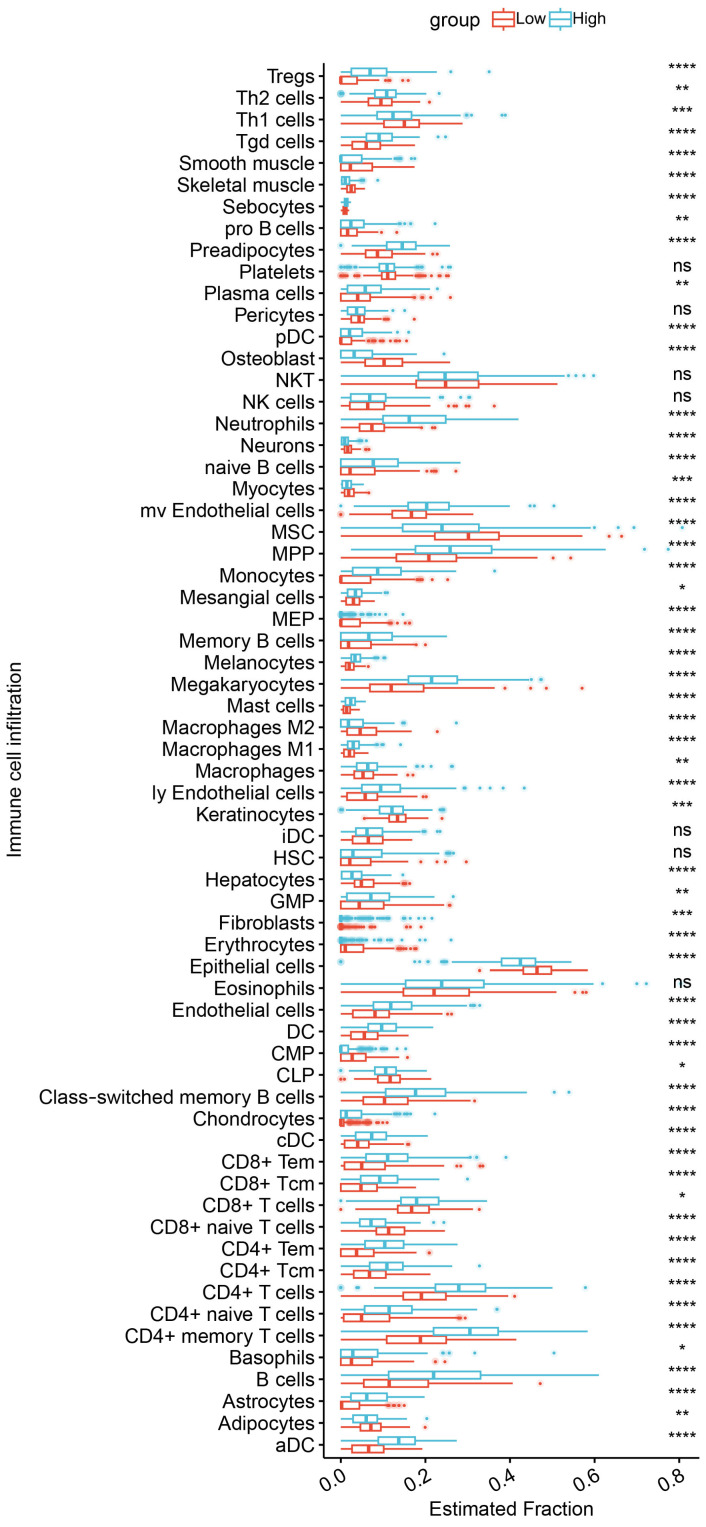
Immune infiltration analysis of pleiotropic genes in IBD cohort. Abundance of differences in immune cells between both groups in IBD cohort. (ns, not significant; * *p* < 0.05, ** *p* < 0.01, *** *p* < 0.001, and **** *p* < 0.0001).

To validate the subtypes of IBD, immunedeconv was used to quantify pleiotropic genes for each patient, and a pleiotropic gene score was created for each patient based on gene expression. Subsequently, the patients were divided into high- and low-immune-infiltration groups based on the median polytropic gene score. A histogram was generated to visualize the distinct immune cell infiltration patterns between the two groups. The proportion of most immune cell types, including activated and central memory CD4 T cells, dendritic cells, CD8-positive T cells, neutrophils, plasma cells, mast cells, and regulatory T cells (Tregs), was higher in the high-risk group. However, the proportion of M2 macrophages was higher in the low-risk group, as shown in Figure 7.

## 4. Discussion

IBD patients often present with co-occurring immune-mediated diseases that share genetic and immunological similarities mainly driven by helper T-cell (Th) response [46,47]. A common feature of these diseases is their immune pathogenesis, characterized by hyperactivity within specific components of the immune system [48,49]. This shared, complex genetic architecture necessitates an integrated analytical approach, rather than a traditional single-disease framework, to identify genetic factors impacting both conditions. In this study, we employed an innovative, comprehensive method to investigate the genetic relationship between IBD and 16 immune-mediated diseases.

GWAS summary data from two IBD datasets were integrated into the consolidated META-IBD dataset. Of the 16 immune-mediated diseases, 4 diseases exhibited a significant genetic correlation with IBD. All of these four diseases, namely, psoriasis, AS, iridocyclitis, and PsA, are extraintestinal manifestations of IBD. These findings provide valuable insights into the treatment of extraintestinal manifestations of IBD. Among these, AS exhibits the greatest genetic overlap with IBD, with a correlation coefficient exceeding 0.5. Previous studies have indicated a positive causal relationship between IBD and its subtypes and AS. Although the mechanisms underlying their comorbidity remain incompletely understood, the “gut-joint axis” hypothesis has been proposed to explain their pathogenic connection [50]. Various environmental factors, such as dysbiosis of the gut microbiota, along with host immune factors, may lead to inflammation in genetically susceptible individuals, triggering inflammatory responses targeting both gut and joint components. These mechanisms provide important insights for understanding the comorbidity between AS and IBD [51]. The META-IBD dataset and the four immune-mediated diseases were subjected to a comprehensive multi-trait analysis aimed at enhancing the statistical scrutiny of the original IBD datasets. The results revealed 909 previously unidentified genetic loci, substantially improving the understanding of the genetic foundation of IBD. Furthermore, COJO and FUMA analyses revealed 28 independent risk loci closely associated with IBD, including 2 loci (rs1990760 and rs1990760) located in exonic regions with CADD scores exceeding 12.37. IFIH1 plays a role in the innate immune system by recognizing viruses and initiating antiviral responses [52]; however, its functional upregulation may modestly increase the risk of autoimmune diseases [53]. A meta-analysis indicated that the IFIH1 rs1990760 T allele is significantly associated with susceptibility to autoimmune diseases, including systemic lupus erythematosus, multiple sclerosis, and rheumatoid arthritis [54]. This mechanism likely involves IFIH1 enhancing IFN-α production to counteract viral infections while concurrently co-operating with IFNAR to activate the JAK-STAT pathway, thereby inducing the transcription of downstream genes and promoting the development of autoimmune diseases (AIDs) [55]. Therefore, the association of rs1990760 with IBD and other immune-mediated diseases warrants further investigation. IL-23 is secreted by activated dendritic cells, monocytes, and macrophages and is crucial for the amplification and maintenance of Th17 cell responses [56]. The IL-23/Th17 axis plays a key role in the inflammatory processes of IBD and psoriasis [57]. Studies have shown that rs11209026, a missense variant (Arg381Gln) located in the cytoplasmic tail of the IL-23 receptor (IL-23R), can alter IL-23R signaling [58]. Specifically, the IL23R polymorphism (rs11209026) has been shown to increase susceptibility to IBD in the Finnish population [59]. Additionally, the rs11209026 variant in IL23R is significantly associated with the risk of IBD, AS, and psoriasis. These findings suggest that the IL-23/Th17 pathway and its key variants play an important role in multiple immune-related diseases. Furthermore, the integrated approach used in this study not only revealed novel genetic loci but also proposed novel insights into the genetic characteristics of IBD. Genetic association analysis revealed 137 genes related to IBD. The SMR analysis further confirmed 14 genes. These include genes considered mature candidates, such as MAP3K8, IL1RL1, IL18R1, and IL18RAP, as well as less common genes like CD6. CD6 is a surface glycoprotein with a weight of 105–130 kDa, primarily expressed in mature T lymphocytes, CD56 NK cells, and some cells of the hematopoietic system [60,61].

A previous study has already identified CD6 as a negative regulatory factor in both early- and late-stage signal transduction events in T-cell responses [62]. Genetic variations in CD6 are associated with susceptibility to or severity of various immune-mediated diseases, including multiple sclerosis, Behçet’s disease, and psoriasis [63,64,65]. Recent research also indicates that CD6 is a novel risk gene for inflammatory bowel disease (IBD). In the inflamed mucosa of active IBD patients, CD6 expression is significantly increased and positively correlated with disease severity [66,67]. CD6 knockout mice exhibit markedly decreased inflammation in experimental immune-mediated encephalomyelitis (EAE), psoriasis, and uveitis. Additionally, Th1 and Th17 cell-mediated immune responses are significantly reduced [68,69]. Additionally, humanized anti-CD6 mAb (Itolizumab) effectively treats psoriasis and rheumatoid arthritis by strongly suppressing pro-inflammatory cytokine production and Th1/17 cell proliferation [70,71]. This suggests that CD6 may be a potential therapeutic target for IBD. MAP3K8 (also known as TPL2) plays a critical role in the immune system, with its genetic polymorphisms associated with the development of IBD [72]. It promotes the migration of neutrophils to inflammation sites and enhances cytokine secretion, leading to colitis in mice [73,74]. Additionally, MAP3K8 positively regulates the MEK1/2-ERK1/2 signaling pathway, influencing the survival and proliferation of intestinal mucosal cells [75]. The inhibition of MAP3K8 has shown therapeutic potential in various inflammatory diseases, including IBD, RA, and psoriasis, highlighting its value as an important therapeutic target [76,77]. Meanwhile, RNASET2 may regulate the downstream secretion of interferon-gamma (IFN-γ) through integrin signaling pathways, positioning it as a potential therapeutic biomarker [78]. PIM3, a member of the PIM kinase family, is significant in regulating T-cell proliferation; inhibiting PIM3 can induce CD4+ T cells to halt in the G0/G1 phase, preventing their proliferation, and PIM3 inhibitors have demonstrated effectiveness in CD4+ T cell-mediated IBD models [79]. Furthermore, IL-18 and its receptors, IL18RAP and IL18R1, are crucial in immune responses, with IL-18 activating the NF-κB pathway to stimulate the production of interferon-gamma and pro-inflammatory cytokines like TNF-α and IL-1β [80]. Clinical studies show significant increases in IL-18 expression in the mucosal biopsies of IBD patients, particularly in affected lesions, suggesting a key role for IL-18 in IBD pathology [81]. Animal studies indicate that blocking IL-18 can effectively reduce intestinal inflammation [82]. Additionally, the TGF-β signaling pathway plays a vital role in promoting regulatory and inflammatory immune responses, with SMAD3, a downstream transcription factor, being essential [83]; mice lacking SMAD3 are prone to inflammation and develop IBD under environmental stimuli, underscoring the importance of this pathway in tissue repair and immune regulation [84,85]. Finally, the NKX2-3 gene has an SNP rs10883365 located 5 kb upstream of its transcriptional start site that correlates with IBD [86], although the exact mechanisms remain unclear and warrant further investigation. These findings provide significant insights into the pathogenesis of IBD and the development of novel therapeutic strategies.

Using the BLISS method, our study identified 514 proteins associated with the risk of IBD immune-mediated diseases from the deCODE, UKBPP, and ARIC databases. Encouragingly, IL1RL1 was found in multiple analyses (MAGMA, POPS, SMR, and BLISS). The ST2 receptor belongs to the interleukin (IL)-1 receptor family, encoded by the IL1RL1 gene [87]. IL-33 is the functional ligand of ST2L, capable of inducing the production of Th2 cell cytokines and enhancing Th1, Th2, and Th17 immune responses [88,89,90]. The IL-33/IL1RL1 pathway plays a crucial role in host defense and in the pathogenesis of immune-mediated, allergic, and chronic inflammatory diseases such as asthma, dermatitis, arthritis, and Alzheimer’s disease [91].

IL1RL1 levels have been reported to be elevated in the intestines of patients with IBD, correlating with disease activity, and both IL1RL1 and IL-33 levels are higher in circulation in these patients [92]. It has been demonstrated that anti-TNF therapy reduces IL1RL1 subtype A levels while increasing soluble subtypes, providing more decoy receptors to sequester IL-33 and mitigate inflammation [93]. This suggests a potential therapeutic target within the IL-1 pathway for managing IBD and related diseases. The significance of IL1RL1 in our findings emphasizes the relevance of the IL-1 pathway, a central component of pro-inflammatory response and signaling [94]. The IL-1 pathway could be a common thread in the development of further immune-mediated diseases in patients with IBD, or at least in a subset of them, on the basis of autoinflammatory mechanisms. From a therapeutic standpoint, diseases successfully treated with IL-1 inhibitors could benefit from this insight. Furthermore, the development of anti-IL-1R1 monoclonal antibodies for rheumatoid arthritis, Still’s disease, and osteoarthritis underscores the potential for targeting this pathway in IBD [95,96]. This could have significant implications for the understanding and treatment of these conditions in the context of IBD.

The immunedeconv R package was used to assess the degree of immune cell infiltration in IBD. The results indicated that the high-immune-infiltration group included not only common immune cell types observed in IBD, such as activated CD4 T cells, central memory CD4 T cells, CD8-positive T cells, plasma cells, and mast cells, but also less common cell types, such as dendritic cells, M1 macrophages, neutrophils, and Tregs, which may be involved in the onset and progression of IBD. The colonic mucosa of patients with IBD has a high infiltration and secretion of CD4 T cell-associated cytokines, which eventually impair intestinal mucosal barrier function [97,98]. Therefore, CD4 T cells play a crucial role in the pathogenesis of IBD. Neutrophils capable of producing reactive oxygen species are one of the causes of a compromised intestinal barrier in IBD [99]. B cells, dendritic cells, and macrophages can induce chronic inflammation specific to IBD through various pathways, including the regulation of immune responses, antigen processing, and the modulation of inflammation [100,101]. Additionally, mast cells, essential for maintaining homeostasis through barrier defense mechanisms, have a significantly high activity in Crohn’s disease [102]. In this study, the proportion of M2 macrophages was higher in the low-immune-infiltration group. Consistently, previous studies have demonstrated that M2 macrophages can promote tissue repair and reduce inflammation, thereby alleviating the symptoms of IBD [103]. Altogether, the findings of this study highlight the importance of using polyfunctional genes in the identification of IBD subtypes and enhance the understanding of the role of various immune cells in the pathogenesis of IBD. Future drug development can be explored at the pleiotropic loci we identified to develop precise IBD drugs. However, this manuscript also has some limitations. First, the GWAS datasets used in this study were derived from individuals of European descent. Therefore, the results lack generalizability and necessitate further research involving diverse ethnic groups. Additionally, while we identified genes associated with IBD and immune-mediated disease comorbidity, the influence of epigenetic modifications on these loci remains unexplored, which could significantly impact gene expression and disease phenotypes. Furthermore, disease subgroups such as early-onset IBD may exhibit distinct genetic and biological characteristics, affecting the applicability of our results across different IBD subtypes. Future longitudinal studies and experimental investigations are essential to clarify these underlying biological mechanisms comprehensively.

## 5. Conclusions

In conclusion, this study highlights the strong genetic relationship between IBD and four immune-mediated diseases, revealing novel genetic risk factors. The findings improve the understanding of the genetic landscape of IBD, which may facilitate the development of innovative treatment approaches.

## Data Availability

The datasets used in this study can be obtained from the corresponding author.

## Supplementary Materials

The following supporting information can be downloaded at: https://www.mdpi.com/article/10.3390/biomedicines12112562/s1, Table S1: Baseline characteristics of the study population; Table S2: Genetic correlation between inflammatory bowel disease (ieu-a-31 and FinnGen database) and 16 immune-mediated diseases; Table S3: 455 independent associations identified with GCTA-COJO (conditional and joint) analysis applied to GWAS summary statistics (MTAG-IBD); Table S4: Pleiotropic genomic loci in IBD and (Ankylosing Spondylitis, Psoriasis, Iridocyclitis and Psoriatic Arthritis) identified by FUMA using PLACO results; Table S5: The annotation in fuma bassed on the result of MAGMA; Table S6: MAGMA combined with POPS screening (POPS score > 1); Table S7: SMR analysis from eQTL to MTAG-IBD (FDR < 0.05 and p_HEIDI > 0.01); Table S8: KEGG pathway and Gene ontology enrichment of pleiotropic genes; Table S9: Mammalian Phenotype ontology based on pleiotropic genes; Table S10: Bliss analysis from pQTL (ARIC, Decode and UKBPP) to MTAG-IBD (FDR < 0.05 and p_HEIDI > 0.01); Table S11: KEGG enrichment analysis of risk proteins; Table S12: Drug-Gene interaction network of the annotated genes through DGIdb; Table S13: Bidirectional Mendelian randomization estimates of the relationship between genetically instrumented inflammatory bowel disease meta and four autoimmune diseases (iridocyclitis, psoriasis, ankylosing spondylitis and psoriatic arthritis); Table S14: Heterogeneity test and Horizontal pleiotropy test.

## Abbreviations

GWAS	Genome-wide association study
LDSC	Linkage disequilibrium score regression
POPS	Polygenic priority score
MAGMA	Multi-marker analysis of genomic annotation
COJO	Conditional and joint analysis
BLISS	Biomarker Imputation from Summary Statistics
GSVA	Gene set variation analysis
SMR	Summary-based Mendelian randomization
MR	Mendelian randomization
IVW	Inverse-variance weighted
SNPs	Single-nucleotide polymorphisms
95% CI	95% confidence interval
AS	Ankylosing spondylitis
PsA	Psoriatic arthritis
IBD	Inflammatory bowel disease
RA	Rheumatoid arthritis
KEGG	Kyoto encyclopedia of genes and genomes
GO	Gene ontology
